# Butyrate Treatment of DSS-Induced Ulcerative Colitis Affects the Hepatic Drug Metabolism in Mice

**DOI:** 10.3389/fphar.2022.936013

**Published:** 2022-07-19

**Authors:** Lenka Jourova, Stefan Satka, Veronika Frybortova, Iveta Zapletalova, Pavel Anzenbacher, Eva Anzenbacherova, Petra Petr Hermanova, Barbora Drabonova, Dagmar Srutkova, Hana Kozakova, Tomas Hudcovic

**Affiliations:** ^1^ Department of Medical Chemistry and Biochemistry, Faculty of Medicine and Dentistry, Palacky University Olomouc, Olomouc, Czechia; ^2^ Department of Pharmacology, Faculty of Medicine and Dentistry, Palacky University Olomouc, Olomouc, Czechia; ^3^ Laboratory of Gnotobiology, Institute of Microbiology of the Czech Academy of Sciences, Novy Hradek, Czechia

**Keywords:** gut–liver axis, butyrate, gut inflammation, drug metabolism, cytochromes P450

## Abstract

The development of inflammatory bowel disease (IBD) is associated with alterations in the gut microbiota. There is currently no universal treatment for this disease, thus emphasizing the importance of developing innovative therapeutic approaches. Gut microbiome-derived metabolite butyrate with its well-known anti-inflammatory effect in the gut is a promising candidate. Due to increased intestinal permeability during IBD, butyrate may also reach the liver and influence liver physiology, including hepatic drug metabolism. To get an insight into this reason, the aim of this study was set to clarify not only the protective effects of the sodium butyrate (SB) administration on colonic inflammation but also the effects of SB on hepatic drug metabolism in experimental colitis induced by dextran sodium sulfate (DSS) in mice. It has been shown here that the butyrate pre-treatment can alleviate gut inflammation and reduce the leakiness of colonic epithelium by restoration of the assembly of tight-junction protein Zonula occludens-1 (ZO-1) in mice with DSS-induced colitis. In this article, butyrate along with inflammation has also been shown to affect the expression and enzyme activity of selected cytochromes P450 (CYPs) in the liver of mice. In this respect, CYP3A enzymes may be very sensitive to gut microbiome-targeted interventions, as significant changes in CYP3A expression and activity in response to DSS-induced colitis and/or butyrate treatment have also been observed. With regard to medications used in IBD and microbiota-targeted therapeutic approaches, it is important to deepen our knowledge of the effect of gut inflammation, and therapeutic interventions were followed concerning the ability of the organism to metabolize drugs. This gut–liver axis, mediated through inflammation as well as microbiome-derived metabolites, may affect the response to IBD therapy.

## Introduction

To get a deeper insight into physiological processes and metabolism in humans, not only our own cells (with their whole genetic and enzymatic equipment) but also the bacterial cells residing in our body should not be forgotten. This varied bacterial community has been shown to contribute significantly to regulating diverse host physiological functions and, moreover, when corrupted, it has also been implicated with various pathologies (Hills et al., 2019). Indeed, chronic diseases ranging from metabolic syndrome to gastrointestinal disorders and even neurodegenerative diseases are of increasing prevalence in Western societies, and a dysbiotic state of the gut microbiota is beginning to be recognized as an important environmental factor here (Fan and Pedersen, 2021). One example of such a disease where the intestinal microbiome plays a crucial role in inflammatory bowel disease (IBD), a chronic relapsing immune-mediated inflammation of the gastrointestinal tract (Franzosa et al., 2019; Glassner et al., 2020; Mentella et al., 2020).

IBD, especially its two most common clinical forms, ulcerative colitis (UC) and Crohn´s disease, has been present in the human population for thousands of years. However, their incidence is increasing dramatically due to the deteriorating lifestyle. Like many complex diseases, the development of IBD is subject to the successive combination of several factors of the genetic, immunological, and physiological background. IBD is often associated with changes in the gut microbiome composition/function (Tlaskalova-Hogenova et al., 2005; Jakubczyk et al., 2020). UC is a mucosal inflammation limited to the colon, characterized by bloody diarrhea, abdominal pain, and ulcers. Unfortunately, approximately half the patients may develop a more complicated course of the disease which is often due to resistance to pharmacotherapy (Singh et al., 2018; Farrell, 2019).

Gut microbiota is not only an important agent during pathophysiological processes but it can also influence pharmacotherapy outcomes by affecting the biotransformation and pharmacokinetics of a wide range of clinically used drugs including the drugs used to treat IBD (Jourova et al., 2016; Jourova et al., 2019; Zemanová et al., 2021). Moreover, the gut microbiome may also indirectly influence the level of drug-metabolizing enzymes through bacterial metabolites. In the last 2 decades, experiments on gnotobiotic animals highlighted the role of microbiome-derived metabolites in the regulation of the expression, interestingly, not only intestinal but also hepatic cytochromes P450 (Selwyn et al., 2015; Jourová et al., 2020a; Jourová et al., 2020b; Zemanová et al., 2020).

Cytochromes P450 (CYPs) are key enzymes involved in the initial drug metabolism in humans (Anzenbacher and Anzenbacherová, 2001). They metabolize most of the xenobiotics including 70%–80% of all drugs in clinical use and significantly contribute to variability in drug response. The expression of CYPs is regulated by specific nuclear receptors such as aryl hydrocarbon receptor (AhR), constitutive androstane receptor (CAR), and pregnane X receptor (PXR) (Zanger and Schwab, 2013).

The most interesting gut microbiome-derived metabolite is unquestionably butyrate; an endogenous compound produced by bacterial fermentation mainly from dietary fiber in the colon. Butyrate has been intensively studied for its role as a histone deacetylases inhibitor, mainly for its protective role in intestinal homeostasis and immune regulation (Bach Knudsen et al., 2018). Although colonic butyrate production can be effectively stimulated by the consumption of dietary fiber, there is a growing number of pharmaceutical dietary supplements based on oral administration of sodium butyrate with the potential to ameliorate clinical symptoms of inflammatory bowel disease (Di Sabatino et al., 2005; Cleophas et al., 2019). Butyrate, however, has an ability to regulate the expression of a wide range of genes, and through circulation it may reach various distal organs far beyond the gastrointestinal tract. As the liver receives most of its blood and nutritional supply from the gut, it may be easily exposed to bacterial components and metabolites such as butyrate. Once in the liver, butyrate can influence liver physiology and metabolism including the metabolism of drugs through induction of nuclear receptors (Jourova et al., 2022). This effect is even more pronounced when the gut barrier is compromised which is one of the main features of IBD (Chang, 2020). As the administration of butyrate into the lumen of the colon is one of the promising approaches in the therapy of UC (Hallert et al., 2003), further studies of the possible effect of butyrate on the hepatic biotransformation enzymes are needed.

We hypothesize that oral administration of sodium butyrate may simultaneously increase the portion of butyrate reaching the liver and directly affect hepatic drug metabolism. Also, butyrate administration may influence hepatic CYPs regulation indirectly, through affecting gut microbiota composition and immune cell differentiation (Lee et al., 2022). We also suggest that the effect of butyrate administration may vary under physiological and inflammatory states as it has been published previously that patients with UC have impaired butyrate metabolism and uptake (De Preter et al., 2012).

For this reason, the aim of our study was to clarify not only the protective effects and underlying mechanisms of the sodium butyrate (SB) administration on colonic inflammation induced by dextran sulfate sodium (DSS), but we focused also on the effects of SB treatment on hepatic drug metabolism in the murine model of inflammation. In our experiment, the two different time settings of butyrate administration have been used, and butyrate has been administered also to healthy mice without induced colitis to evaluate the difference.

## Materials and Methods

### Animals and Experimental Design

Two-month-old BALB/c mice (females) reared in SPF (specific-pathogen-free) conditions were used. Mice were fed with 25 kGy irradiated sterile pellet diet (Altromin, Lage, Germany) and drinking water *ad libitum*. The animals were kept in a room with a 12 h light/dark cycle at 22°C. SPF mice were regularly checked for the absence of potential pathogens according to an internationally established standard (FELASA). The mice were divided into five groups (five mice per group) and treated with sodium butyrate (SB; M.W. 110.09 g/mol; Sigma-Aldrich, St. Louis, United States) (Vieira et al., 2012) and/or dextran sulfate sodium (DSS, M.W. 36–50 kDa; MP Biomedicals, Illkirch, France). DSS was dissolved in water to obtain 2.5% solution and autoclaved (Hudcovic et al., 2001). The 0.5% SB (w/v) was prepared in drinking water (groups 2 and 4) or in 2.5% DSS (group 3) and filtered. Both solutions were refreshed regularly every day. Control groups received drinking water only (group 1) or 2.5% DSS solution (group 5) (Table 1). The experiment was repeated twice and representative data from one experiment are shown.

**TABLE 1:** Experimental design.

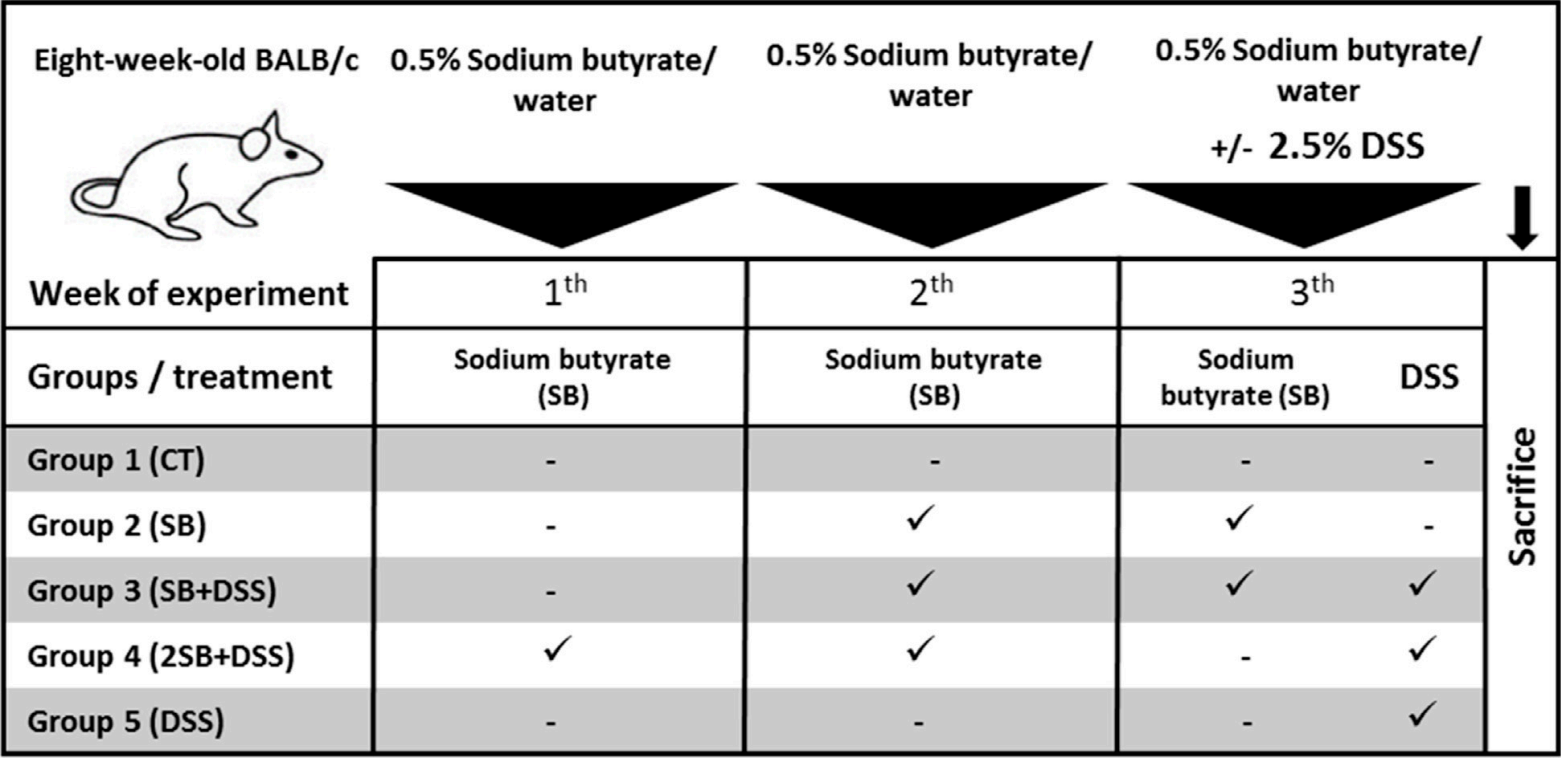

All procedures with animals were approved by the Ethics Committee, Ministry of Education of the Czech Republic. Experiments were approved by the Committee for the Protection and Use of Experimental Animals of the Institute of Microbiology, v. v. i, Czech Academy of Sciences of the Czech Republic (approval ID: 21/2018).

During experiments, the health status of mice was monitored every day. At the end of the experiments, the mice were euthanized by isoflurane (IsoFlo, Zoetis CZ, Prague, Czech Republic) followed by cervical dislocation. The blood was collected in tubes with ethylenediaminetetraacetic acid (EDTA) as an anticoagulant, centrifuged, and the plasma was stored at −80°C before being assayed. The clinical symptoms of colitis were evaluated as: occurrence of diarrhea, rectal prolapses, and rectal bleeding with scoring: 0—no diarrhea or rectal bleeding; 1—pasty stool or mild, no visible rectal bleeding, positive hemocult test; 2—diarrhea or loss of feces, mild visible blood in feces or anus; 3—extensive bleeding from the rectum, rectal prolapses. The colon was aseptically removed, length was measured and individual segments were processed for histological analyses or frozen in liquid nitrogen and stored at −80°C. The livers were aseptically removed, weighed, frozen in liquid nitrogen, and subsequently stored at −80°C until further processing.

### Histological Evaluation of Inflammation

The tissue was fixed in Carnoy’s fluid for 30 min, transferred into 96% ethanol, and embedded in paraffin. Five μm paraffin-embedded sections were cut and stained with hematoxylin and eosin (H&E), or Alcian Blue, with post-staining by Nuclear Fast Red (all from Vector Labs, Burlingame, United States) for mucin production. The samples were viewed under Olympus BX 40 microscope equipped with an Olympus Camedia DP 70 digital camera, and the images were analyzed using Olympus DP-Soft. The degree of damage to the surface epithelium, crypt distortion, and mucin production in individual colon segments was evaluated according to (Cooper et al., 1993; Hudcovic et al., 2001).

### Immunohistochemical Evaluation of Zonula Occludens-1 (ZO-1) Expression in Colon

Colon-descendent segments were embedded in O.C.T. Tissue-tek (Sakura Finetek, Alphen aan den Rinj, NL) and were frozen in liquid nitrogen. Acetone-fixed cryosections (5 μm thick) were used for immunohistochemistry. Expression of ZO-1 was detected by rabbit anti-mouse polyclonal antibody (Zymed/Thermo Fisher, San Francisco, United States), and secondary antibody Cy3 goat anti-rabbit IgG (Biomeda, Foster City, United States). The samples were viewed under Olympus BX 40 microscope equipped using an Olympus Camedia DP 70 digital camera, and the images were analyzed using Olympus DP-Soft.

### Western Blot Analysis of ZO-1

Western blot analysis was done according to Schwarzer et al., (2016). Frozen colon samples were homogenized in T-PER (tissue protein extraction, Thermo Scientific, IL, United States) supplemented with protease inhibitors (Halt™ Protease and Phosphatase Inhibitor Cocktail, Thermo Scientific, IL, United States). After centrifugation of the lysates, protein concentration was estimated in the supernatant using a PierceTM BCA Protein Assay Kit (Thermo Scientific, IL, United States). Total proteins (4 µg/µl) were separated on 4%–20% precast gels SDS–polyacrylamide gel (Bio-Rad Laboratories, United States) and then transferred to Immobilon-P membranes (PVDF membrane, Merck Millipore, MA, United States). Membranes were blocked with 4% BSA in TBS-Tween for 1 hour at RT before being probed overnight at 4°C with specific primary antibodies against ZO-1 (Invitrogen, CA, United States). After washing, Ab binding was revealed by incubation with anti-rabbit IgG HRP-conjugated antibody (Abcam, Cambridge, United Kingdom) for 1 hour at room temperature, and proteins were visualized by chemiluminiscent (Immobilon Crescendo Western HRP Substrate, Merck Millipore, Germany) substrate according to the manufacturer’s instructions. Signals were measured by C-Digit Blot Scanner (LI-COR, Lincoln, United States) and quantified using the Image Studio Digits, version 3.1 and program ImageJ.

### Cytokine Evaluation in Plasma

The concentration of IFN-γ, IL-6, and IL-10 cytokines was determined in the plasma of experimental mice by the MILLIPLEX MAP Mouse Cytokine/Chemokine Panel (Merck Millipore, MA, United States) according to the manufacturer’s instructions and analyzed using the Bio-Plex System (Bio Rad Laboratories, CA, United States).

### Preparation of Microsomal Fraction

Murine livers were pooled (5 animals per group) into individual groups (according to Table 1). Microsomal fractions were prepared by differential centrifugation as mentioned elsewhere (Prokop et al., 2018). Samples of liver tissue homogenate and final microsomal fractions were stored at −80°C. Concentrations of cytochrome P450 in liver microsomes were determined spectrophotometrically using carbon monoxide (Schenkman and Jansson, 2006).

### RNA Isolation and Quantitative Real-Time RT-PCR (qRT-PCR)

Total RNA was isolated from murine liver tissues stored in RNAlater using an RNeasy Plus Mini Kit (Qiagen, Hilden, Germany) following the manufacturer’s protocol. RNA concentration and purity were quantified spectrophotometrically at 230, 260, and 280 nm using a NanoPhotometer N60 (Implen, Munich, Germany). One µg of total RNA was converted to mRNA using a Transcriptor High Fidelity cDNA synthesis kit (Roche, Basel, Switzerland) according to the manufacturer’s instrumentations. The qPCR was performed in a LightCycler 1536 Instrument (Roche, Basel, Switzerland) using specific TaqMan Gene Expression Assays (Applied Biosystems Waltham, Massachusetts, United States). The 1536-well plates were pipetted using Echo Liquid Handler (Labcyte, Dublin, Ireland). The expression of the following genes *Cyp1a1*, *Cyp1a2*, *Cyp2b10*, *Cyp2c38*, *Cyp3a11*, *Cyp3a13*, and *Il-1β* was normalized to the expression of the housekeeping gene hypoxanthine guanine phosphoribosyl transferase (*Hprt*). Expressions of the target genes were calculated by the ΔΔCT method (Livak and Schmittgen, 2001) as a fold change in the treatment groups relative to the control group 1.

### Cytochrome P450 Enzyme Activity Assays

Enzyme activities were measured in the mouse hepatic microsomal fractions according to the established methods (Kronbach et al., 1989; Phillips and Shephard, 2006). For the determination of murine CYP activities which are orthologues of human liver drug-metabolizing CYPs, the following substrates were used: CYP1A1/CYP1A2: 7-ethoxyresorufin (Santa Cruz Biotechnology, Heidelberg, Germany); CYP2B: O-penthoxyresorufin (Toronto Research Chemicals, Canada); CYP2C: diclofenac (Sigma-Aldrich, St. Louis, United States) and CYP3A: midazolam (Abcam, Cambridge, United Kingdom). Metabolites 7-hydroxycoumarin (Santa Cruz Biotechnology, Heidelberg, Germany) and 1′-hydroxymidazolam (Cayman Chemical, Michigan, United States) were used as controls. Incubation mixtures contained 100 mM potassium phosphate buffer (pH 7.4), hepatic microsomes, substrates, and NADPH-generating system consisting of isocitrate dehydrogenase, NADP+, isocitrate, and MgSO_4_ (Sigma-Aldrich, Co., St. Louis, United States). Detailed descriptions of enzyme activity assays are summarized in Table 2. Activities of murine CYPs were measured using a Shimadzu LC-20 HPLC system (Shimadzu, Kyoto, Japan) with UV or fluorescence detection. The measurements were performed in a LiChrospher RP-18 column, or a Chromolith^®^ High Resolution RP-18 endcapped column (determination of midazolam metabolite) (Merck, Germany). All experiments were performed in triplicate and repeated at least twice.

**TABLE 2:** Conditions and HPLC parameters for cytochrome P450 enzyme activity assays.

CYP	Substrate	Metabolite	Concentration of the substrate (µM)	pmol of CYP/Μl	Elution	Detection
1A1/2	7-Ethoxyresorufin	Resorufin	2.6	35/100	Isocratic	Fluorescence
2B	O-Penthoxyresorufin	Resorufin	2.6	35/100	Isocratic	Fluorescence
2C	Diclofenac	4′-OH-Diclofenac	16	35/200	Binary gradient	UV
3A11/13	Midazolam	1′-OH-Midazolam	2.8	12.56/100	Isocratic	UV

### Statistical Analysis

The normal distribution of data was tested using the Shapiro–Wilk test. Statistical evaluation was performed by Student’s paired two-tailed *t*-test and the one-way ANOVA with Tukey’s *post hoc* test using Statistica 12 software (Dell, obtained from StatSoft, Texas, United States). Data are expressed as mean ± SD. Differences were regarded as statistically significant when the *p*-value was lower than 0.05. The number of experiments and replicates are given in each figure legend.

## Results

### Effects of Butyrate on Intestinal Inflammation and Mucosa Damage in DSS-Induced Colitis

To determine the prophylactic effect of butyrate *in vivo*, the mouse model of acute ulcerative colitis induced by administration of 2.5% DSS solution was used. The animals were divided into 5 groups according to Table 1. Healthy control mice (group 1, **CT**, *n* = 5) were without any treatment, and group 2 received sodium butyrate (SB) for 2 weeks (group 2, **SB,** *n* = 5). In group 3, 1-week SB administration was followed by 1-week SB mixed with DSS solution (group 3, **SB + DSS,** *n* = 5). In group 4, SB was administered for 2 weeks at the “preventive” setting and subsequently experimental colitis was induced by 1 week DSS administration (group 4, **2SB + DSS,** *n* = 5). Finally, DSS mice (group 5, **DSS**, *n* = 5) were without SB treatment only with induced colitis by 1 week of DSS administration (Table 1).

**FIGURE 1:**
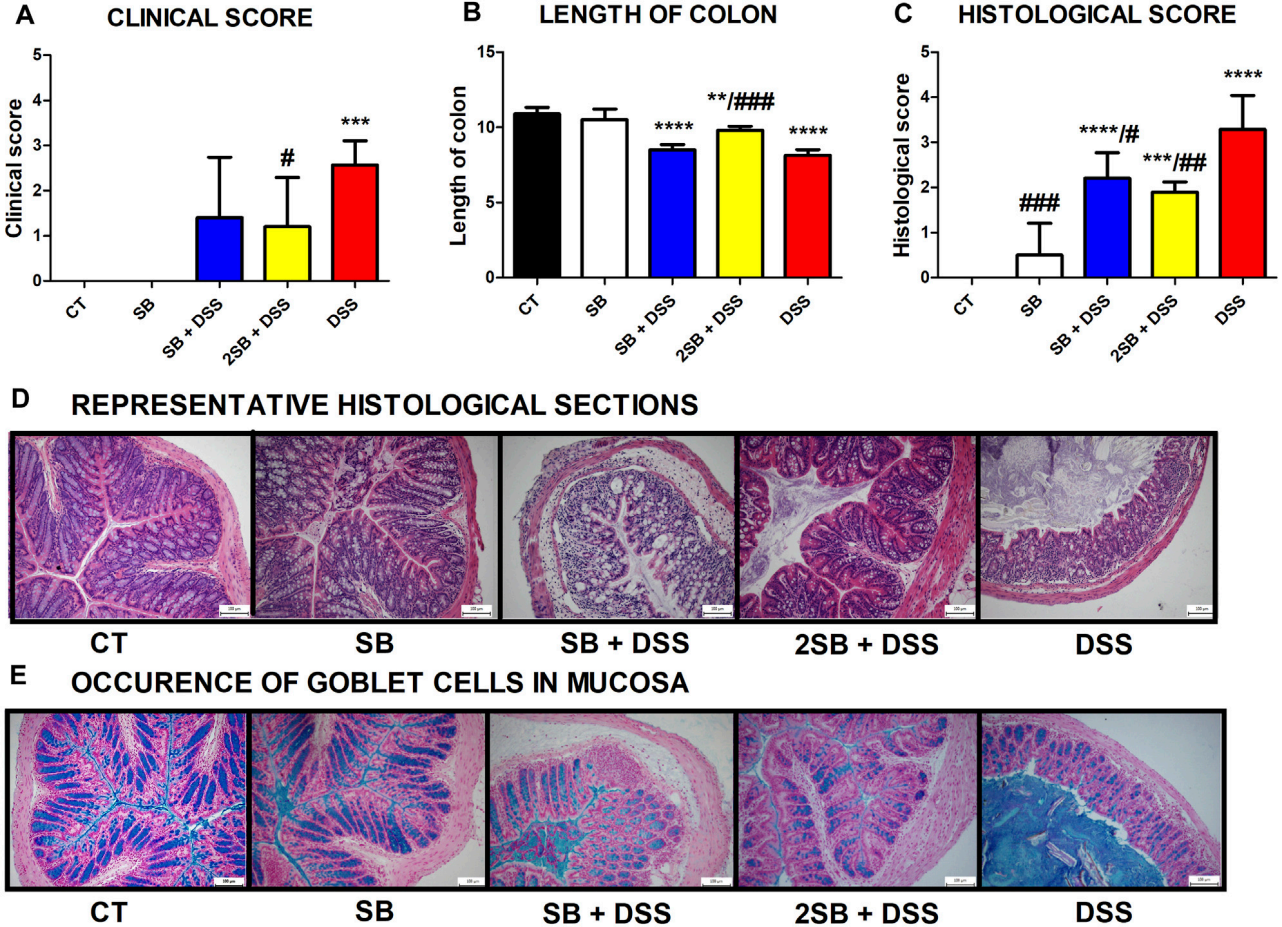
Impact of butyrate pre-treatment on clinical symptoms and histopathological changes in the dextran sodium sulfate mouse model of ulcerative colitis. Altogether 25 mice were equally separated into five groups according to sodium butyrate (SB) and DSS treatment. **(A)** Occurrence of diarrhea and rectal bleeding was summarized in the clinical score. **(B)** Shortening of colon length and **(C)** histopathological changes in colonic mucosa after DSS treatment summarized as the histological score was analyzed at the end of experiment. Changes in colonic mucosa after DSS-treatment are shown on representative histological sections stained by hematoxylin/eosin. **(D)** Occurrence of goblet cells in mucosa of representative colon sections was visualized by Alcian blue/nuclear fast red staining. Data in graphs are expressed as mean ± SD and represent one out of two experiments. One-way ANOVA with Tukey’s multiple *post hoc* test was used for comparison of experimental groups to CT controls (group 1: *p < 0.05, ***p < 0.001, ****p < 0.0001) or to DSS-controls (group 5: ♯*p* < 0.05, ♯♯*p* < 0.001, ♯♯♯♯*p* < 0.0001). **CT**: drinking water; **SB**: 0.5% sodium butyrate in drinking water for 2 weeks; **SB + DSS**: 0.5% sodium butyrate in drinking water 1 week before and the next week together with 2.5% DSS in drinking water; **2SB + DSS**: 0.5% sodium butyrate in drinking water 2 weeks before 2.5% DSS in drinking water; **DSS**: 1 week 2.5% DSS in drinking water.

Disease progression was characterized by the appearance of diarrhea or loose feces and visible rectal bleeding, and summarized as clinical score (0–3) in Figure 1A. DSS administration significantly increased clinical scores and reduced the colon length of mice in group 5 in comparison with control animals (group 1) (Fig. 1AB). Mice pre-treated by 2-week administration of SB prior to DSS (group 4) showed significant improvement in clinical signs of inflammation and reduction of DSS-induced colon shortening compared to DSS-control mice (Fig. 1AB). In contrast, 1 week pre-treatment by SB followed by 1 week DSS + SB mixture administration (group 3) had a weaker impact on these parameters (Figures 1A,B). Butyrate administration itself (group 2) did not affect clinical scoring and colon length in comparison with the control group.

The histological findings showed massive infiltration of inflammatory cells into lamina propria, thickening of submucosa, loss of epithelial layer, and disappearance of the mucosal crypt in the colonic wall of DSS-treated controls (group 5; grade 3.3 ± 0.7), and in weaker levels also in mice with 1 week pre-treatment by SB (group 3; grade 2.2 ± 0.6) (Figures 1C,D). In contrast, 2 week pre-treatment of mice by SB displayed inhibitory effect on DSS-induced histological changes (group 4; grade 1.9 ± 0.2) compared to DSS controls. These mice exhibited reduced infiltration of inflammatory cells and pathological changes in mucosa or epithelial layer. On the other hand, the amount of mucin-producing goblet cells in lamina propria (Alcian blue staining) was decreased in DSS-control mice only (Figure 1E). As previously shown, butyrate can improve epithelial barrier function by stimulating mucin production (Hatayama et al., 2007). In line with these results, it was observed that administration of butyrate solution added significant protection to the maintenance of functional goblet cells producing mucin (Figure 1E).

### Effect of Sodium Butyrate on Tight-Junction Protein Zonula Occludens-1

**FIGURE 2:**
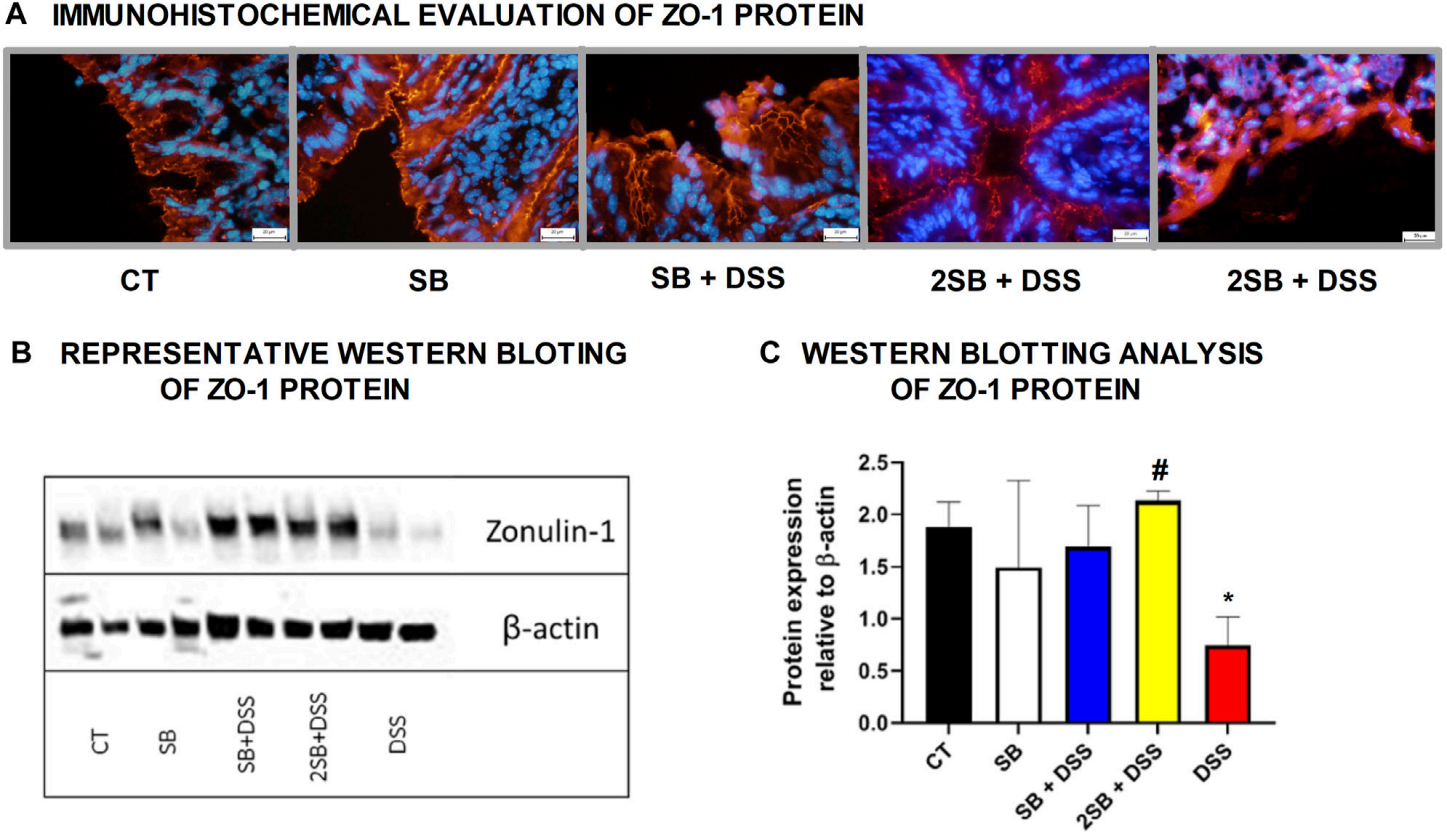
Sodium butyrate administration restored zonula occludens-1 (ZO-1) production in the colon of DSS-treated mice. Effect of butyrate and DSS treatment on production of tight-junction protein ZO-1 was evaluated in cryosections of colonic mucosa of experimental mice. **(A)** Immunohistochemical evaluation was detected using monoclonal antibody ZO-1/CY3 and representative images are shown. **(B)** Representative out of three separated Western blotting assays of ZO-1 protein in tissue of colon is shown. Expression of β-actin was used as an internal control. **(C)** Quantification of the signals was performed using ImageJ, and data are expressed as the mean ± SD from two separated measurements (2 mice per each group), Student’s paired two-tailed *t*-test was used for comparison of experimental groups to CT controls (group 1: *p < 0.05) or to DSS-controls (group 5: ♯*p* < 0.05). **CT**: drinking water for 1 week; **SB**: 0.5% sodium butyrate in drinking water for 2 weeks; **SB + DSS**: 0.5% sodium butyrate in drinking water 1 week before and next week together with 2.5% DSS in drinking water; **2SB + DSS:** 0.5% sodium butyrate in drinking water 2 weeks before and the next week together with 2.5% DSS in drinking water; **DSS:** 1 week 2.5% DSS in drinking water.

The mechanism of butyrate activity which supports the integrity of the intestinal barrier is mediated through promoting the reassembly of tight junctions (TJs) (Peng et al., 2009; Miao et al., 2016). In our experiment, we have shown by immunohistochemistry staining (Figure 2A) and Western blotting (Figures 2B,C) that the production of TJs protein Zonula occludens-1 (ZO-1) was markedly reduced only in DSS-control mice (group 5) compared to control mice (group 1) with intact production of ZO-1. In contrast, administration of sodium butyrate preserved the loss of production and alteration of the distribution of ZO-1 protein (Fig. 2ABC) which was apparent in DSS controls.

### Effect of Sodium Butyrate Treatment on Inflammatory Cytokine Response in Liver and Plasma of DSS-Treated Mice

**FIGURE 3:**
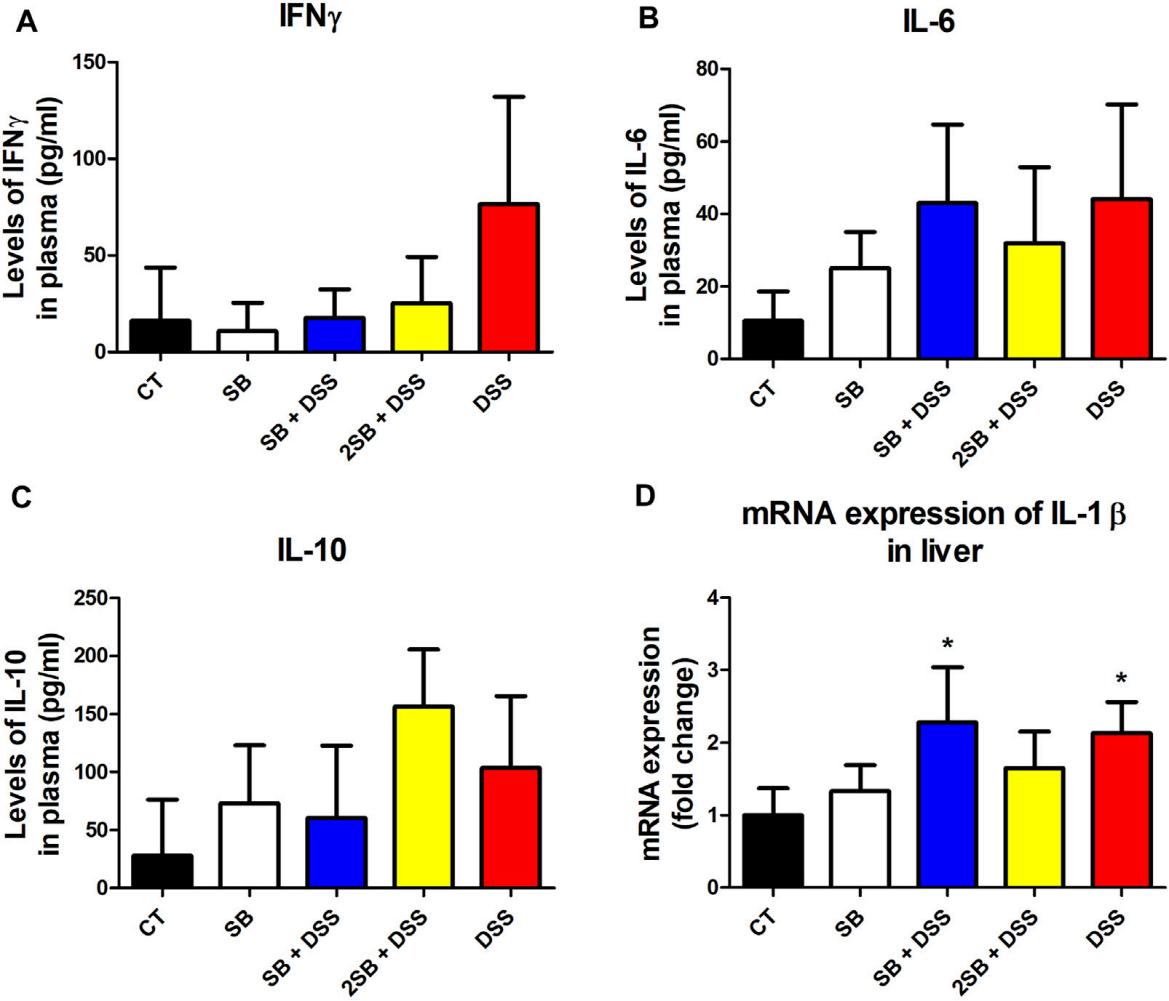
Sodium butyrate administration influence the level of DSS-induced cytokine response in plasma and liver of experimental mice. **(A)** IFN-γ, **(B)** IL-6, and **(C)** IL-10 cytokine levels were measured by ELISA in plasma of experimental mice and are expressed as pg/ml. **(D)** Quantitative PCR analysis of mRNA expression of IL-1β was performed in the liver of mice. Values are expressed as mean ± SD (*n* = 5) and represent one out of two experiments. One-way ANOVA with Tukey’s multiple *post hoc* test was used for comparison of experimental groups to CT controls (group 1: *p < 0.05). **CT**: drinking water for 1 week; **SB**: 0.5% sodium butyrate in drinking water for 2 weeks; **SB + DSS**: 0.5% sodium butyrate in drinking water 1 week before and the next week together with 2.5% DSS in drinking water; **2SB + DSS**: 0.5% sodium butyrate in drinking water 2 weeks before 2.5% DSS treatment in drinking water; **DSS**: 1 week 2.5% DSS in drinking water.

The level of pro-inflammatory cytokine IFN-γ, IL-6, and anti-inflammatory IL-10 was evaluated in the plasma of experimental mice (Fig. 3ABC). In line with macroscopic and histological analyses, DSS-control mice (group 5) exhibited the highest level of pro-inflammatory cytokines IFN-γ and IL-6 compared to healthy controls, although the changes were not statistically significant. Moreover, the administration of sodium butyrate (groups 3 and 4) has shown the tendency to decrease the DSS-induced IFN-γ production in the plasma of mice affected by DSS treatment (Fig. 3BC). Interestingly, 2 weeks administration of SB prior to DSS treatment (group 4) caused the highest increase in the level of anti-inflammatory cytokine IL-10, however again, without statistical significance (Figure 3C).

It has been previously reported that lipopolysaccharides from inflammatory sites in the colon induce hepatic inflammation in DSS-induced murine colitis (Kusunoki et al., 2014). In order to determine induced inflammatory response also in the liver of mice, we evaluated the expression of pro-inflammatory cytokine IL-1β in liver tissue. In our experiment, mRNA expression of IL-1β was increased in the liver of the DSS group of mice (group 5) compared to the control group (group 1) (Figure 3D). A similar increase in mRNA IL-1β expression was observed in group SB + DSS (group 3) compared to control. On the other hand, mRNA IL-1β expression did not reach any significant statistical difference in the group treated with butyrate alone (SB), and interestingly, also in the 2SB + DSS (group 4) in comparison with control mice (Figure 3D).

### Butyrate Treatment of the Murine Model of UC Affects the Expression of Hepatic Cytochromes P450

The expression of selected hepatic CYPs has been determined in the DSS-induced ulcerative colitis female murine model with a focus on the effect of treatment by butyrate. In the following real-time qPCR experiment, we concentrated on the mRNA expression of CYPs from families 1, 2, and 3 (crucial in drug metabolism in humans), namely, Cyp1a1, Cyp1a2, Cyp2b10, Cyp2c38, Cyp3a11, and Cyp3a13. The amount of mRNA is quantified as a relative expression to the level of control mice (group 1).

**FIGURE 4:**
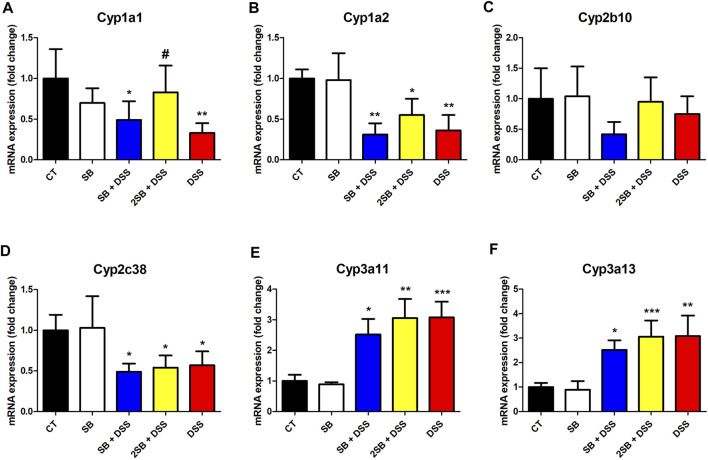
Quantitative PCR analysis of selected hepatic cytochromes P450 **(A)** Cyp1a1, **(B)** Cyp1a2, **(C)** Cyp2b10, **(D)** Cyp2c38, **(E)** Cyp3a11, and **(F)** Cyp3a13 mRNA expressions in the liver of mice. Values are expressed as mean ± SD (*n* = 5). One-way ANOVA with Tukey’s multiple *post hoc* test was used for comparison of experimental groups to controls; * significance to CT (*p < 0.05, **p < 0.01, ***p < 0.001) and # significance to DSS (#*p* < 0.05). **CT:** drinking water for 1 week; **SB:** 0.5% sodium butyrate in drinking water for 2 weeks; **SB + DSS:** 0.5% sodium butyrate in drinking water 1 week before, and the next week together with 2.5% DSS in drinking water; **2SB + DSS:** 0.5% sodium butyrate in drinking water 2 weeks before, and the next week together with 2.5% DSS in drinking water; **DSS:** 1 week 2.5% DSS in drinking water.

Mice treated with DSS have shown significantly decreased mRNA expression of Cyp1a1, Cyp1a2, and Cyp2c38 (Figures 4A,B,D). On the other hand, DSS-induced colitis led to a significant increase of Cyp3a11 and Cyp3a13 mRNA expression in female murine liver (Figures 4E,F).

The following findings were made by analyzing the groups SB + DSS and 2SB + DSS where DSS-induced colitis was treated with butyrate administration in comparison with the DSS group. Only the mRNA expression of Cyp1a1 in liver samples of the mice pre-treated by a 2 week administration of SB prior to DSS (group 4) has shown a significant increase compared to group 5 with DSS-induced colitis (Figure 4A). In the case of Cyp1a2, Cyp2c38, Cyp3a11, and Cyp3a13 the butyrate treatment (groups 3 and 4) did not significantly change the effect of DSS treatment (Fig. 4BDEF). Finally, butyrate applied to a healthy control group (group 2) did not affect the mRNA expression of selected CYPs (Figure 4).

### Butyrate Treatment of the Murine Model of UC Affects Enzyme Activity of Hepatic Cytochromes P450

**FIGURE 5:**
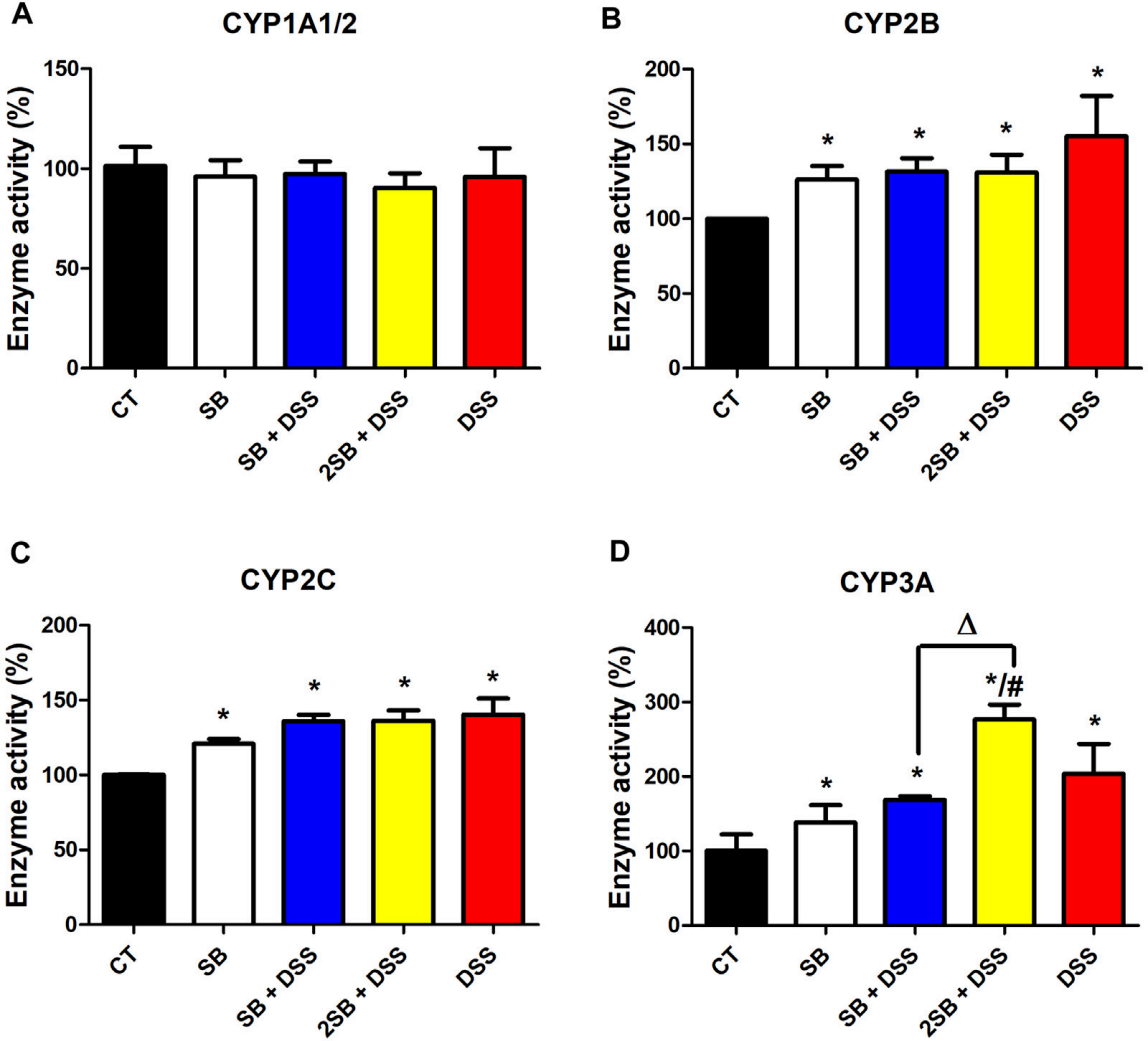
Enzyme activity of selected hepatic cytochromes P450. Enzyme activities of cytochromes P450 **(A)** CYP1A1/2, **(B)** CYP2B, **(C)** CYP2C, and **(D)** CYP3A were measured in the mouse hepatic microsomal fractions using the HPLC system with UV or fluorescence detection. Data represent the mean ± SD from three separated assays measured in pooled microsomal samples of five mice from each group. One-way ANOVA with Tukey’s multiple *post hoc* test was used for comparison of experimental groups to controls; * significance to CT (*p < .05), # significance to DSS (#*p* < .05), and ∆ significance of group 3 to group 4 (∆*p* < .05). **CT**: drinking water for 1 week; **SB**: 0.5% sodium butyrate in drinking water for 2 weeks; **SB + DSS**: 0.5% sodium butyrate in drinking water 1 week before and the next week together with 2.5% DSS in drinking water; **2SB + DSS:** 0.5% sodium butyrate in drinking water 2 weeks before and the next week together with 2.5% DSS in drinking water; **DSS:** 1 week 2.5% DSS in drinking water.

Enzyme activity of CYP2B, CYP2C, and CYP3A was significantly increased in mice where DSS-colitis was induced (groups 3, 4, 5) compared to the control group (Figures 5B,C,D). Treatment with DSS and butyrate in both the time settings (groups 3 and 4) caused an increase in enzymatic activity of CYP2B and CYP2C compared to the control mice (group 1), while the increase was less pronounced than in the case of DSS-treated mice (Figures 5B,C). On the other hand, the activity of CYP3A varied significantly between the groups where DSS-induced colitis was treated by butyrate. Butyrate administered 1 week prior to and 1 week together with DSS solution (group 3) has caused a significant increase in the CYP3A4 activity compared to the control group, similarly to the cases of CYP2B and CYP2C. Surprisingly, butyrate administrated 2 weeks prior to DSS-induced colitis (group 4) has shown an even higher level of CYP3A enzyme activity than the group treated with DSS alone (Figure 5D). Also, the administration of butyrate itself to control “healthy” mice increased the enzyme activity of CYP2B, CYP2C, and CYP3A by about 20%–30%. Finally, the enzyme activity of CYP1A1/2 did not exhibit any changes due to inflammation and/or butyrate treatment compared to control animals (Figure 5A).

## Discussion

There is still no universal cure for IBD (if even possible), and the efficacy of anti-IBD drugs has also been shown to vary among patients (Bergan et al., 1981; Latteri et al., 2001). Moreover, IBD patients often experience extra-intestinal manifestations of IBD such as arthritis and life-threatening comorbidities such as cardiovascular disease, neuropsychological disorders, and colon cancer leading inevitably to further medication (Argollo et al., 2019). Currently, 5-aminosalicylic acid and its derivatives, steroids, and immunosuppressants are used to treat UC. However, the goal of therapy is only to induce and maintain remission and, thus, a safe and effective approach to treat UC has yet to be discovered. As mentioned earlier, gut microbiome-derived metabolite butyrate with its well-known anti-inflammatory effect in the gut is an auspicious candidate. As chronic inflammation is the main feature of IBD, it is, moreover, suggested that butyrate insufficiency is directly linked with colitis (Meisel et al., 2017). The hypothesis is supported by the studies where butyrate has been used to treat colitis with beneficial effects in rodent models (Vieira et al., 2012) or in patients (Scheppach et al., 1992; Vernia et al., 1995). For this reason, it seems that one of the promising approaches in the therapy of IBD is the administration of butyrate into the lumen of the colon.

In the present study, the protective effect of butyrate administration was evaluated using the murine model of acute colitis which was induced by the drinking of 2.5% DSS solution in water for seven consecutive days according to our well-established model (Hudcovic et al., 2012). As expected, significantly shortened colon length and increased clinical and histological scores – typical indicators of colitis severity – were observed in mice treated with DSS (group 5). Butyrate administrated 2 weeks prior to DSS-induced colitis (group 4) led to amelioration of macroscopic and histological signs of inflammation in our experimental design. Interestingly, butyrate administered 1 week prior to and 1 week together with DSS solution (group 3) had no or a weaker effect on inflammatory parameters (Figure 1).

Another main characteristic of IBD in humans is increased intestinal permeability and reduced expression of tight-junction proteins (Pastorelli et al., 2013) which leads to exposure of luminal antigens to the lamina propria (Schulzke et al., 2009). Therefore, several proposed therapeutic approaches to treat IBD are focused on enhancing/restoring gut barrier integrity (Hamer et al., 2010; Gill et al., 2018). The molecular basis of butyrate effect on the assembly of tight-junction proteins has been characterized using different cell line models (Peng et al., 2009; Yan and Ajuwon, 2017). In an experimental model of colitis, administration of butyrate-producing bacterium *Clostridium tyrobutyricum* or *Faecalibacterium prausnitzii* have been shown to restore gut barrier integrity by modulation of tight-junction proteins (Hudcovic et al., 2012; Martín et al., 2015). We have shown that butyrate pre-treatment restored the assembly of tight-junction protein Zonula occludens-1 (ZO-1) in mice with induced colitis and thus reduced the leakiness of colonic epithelium. Here, both the time settings of butyrate application (groups 3 and 4) preserved the loss of production of ZO-1 protein to the same range (Figure 2).

Taken together, the results shown here indicate that butyrate may have a protective effect on the DSS-induced gut inflammation in mice; however, also inconsistent effects of butyrate intervention in murine models of colonic inflammation have been reported in the literature. For example, butyrate enemas did not prevent intestinal damage in TNBS-induced colitis in rats (Tarrerias et al., 2002), while butyrate administration reduced colonic mucosal damage in DSS-treated mice (Ji et al., 2016). In the work of Chang et al. (2014), it has been also shown that butyrate gavage did not revert/prevent DSS-induced intestinal damage in C57BL/6 mice exposed to antibiotics. These contradictory effects of butyrate might be species-specific due to the colitis model (DSS vs. TNBS), commensal bacteria depletion, butyrate dosing, and route of administration. Moreover, in our study, the different administration times of butyrate solution in the preventive setting led to different outcomes in mice with DSS-induced colitis.

It should also be noted that most studies were performed *in vitro* or using rodent models of gut inflammation and the exact molecular mechanism of butyrate action during gut inflammation has yet to be understood. Similarly, several clinical studies proved the alleviation of abdominal symptoms in UC patients (Scheppach et al., 1992; Vernia et al., 1995; Lührs et al., 2002; Hallert et al., 2003); however, the contradictory or minor effect of butyrate enemas applied to UC patients have been shown as well (Steinhart et al., 1996; Hamer et al., 2010). Therefore, to move forward, we need well-designed clinical trials exploring the appropriate dose of butyrate and the role of other factors in the lead with the microbiome in different IBD subtypes.

The effect of butyrate in the gut is still not completely understood; moreover, butyrate is a perspective molecule as it may exert potentially useful effects on many inflammatory and metabolic conditions beyond GIT (Canani et al., 2011). Increased permeability of the gut barrier (a typical feature of IBD) leads to increased concentration of circulating microbiome-derived metabolites in the blood which may reach distal organs including the liver. Indeed, recent studies have reported that gut-derived products may alter hepatic metabolism and stimulate inflammation in the liver (Ding et al., 2019). It is known that gut inflammation induces inflammation also in the liver of UC patients (Hashimoto et al., 1993) and of mice with DSS-induced colitis mainly through LPS translocation (Gäbele et al., 2011; Kusunoki et al., 2014). In our study, the increased level of the inflammatory cytokines in the plasma (Figures 3A,B,C) and mRNA expression of IL-β in the liver of DSS-treated mice was observed (Figure 3D). Although the results did not reach statistical significance, we have observed a tendency to decrease levels of pro-inflammatory cytokines IFN-γ and IL-6 in plasma in the butyrate-treated groups compared to the DSS group (Figures 3A,B). On the other hand, butyrate administrated 2 weeks prior to DSS-induced colitis (group 4) tended to increase the plasmatic level of anti-inflammatory cytokine IL-10 compared to the DSS group (Figure 3C). These data are supported by previous findings demonstrating the beneficial effects of butyrate on murine models of steatohepatitis protecting mice against high-fat diet-induced liver inflammation, fat accumulation, and development of non-alcoholic fatty liver disease (NAFLD) (Mattace Raso et al., 2013; Jin et al., 2016; Zhou et al., 2017; Baumann et al., 2020). Hepatic inflammation causes impaired drug metabolism and is considered, in addition to gene polymorphism, to be a cause of pharmacokinetic variability (Schmith and Foss, 2010). The disruption of drug metabolism is caused mainly by the inhibitory effect of pro-inflammatory cytokines on the expression level and activity of CYPs (reviewed in (Christmas, 2015)). Several hepatic CYPs have been shown to be downregulated in a DSS-induced colitis mice model (Kawauchi et al., 2014; Kusunoki et al., 2014; Fan et al., 2020). Moreover, our recent *in vitro* study has shown that butyrate interferes with hepatic drug metabolism through its effect on the AhR signaling pathway inducing CYP1A1/2 expression and enzyme activity in hepatic cells (Jourova et al., 2022). Therefore, we studied whether administration of butyrate in the two different time settings alongside inflammation can change the expression and enzyme activity of selected hepatic CYPs in mice. Decreased mRNA expression of Cyp1a1 and Cyp1a2 in mice treated with DSS compared to the control group were observed (Figures 4A,B). Administration of butyrate did not affect these trends with one exception – butyrate application to group 4 restored the level of Cyp1a1 mRNA expression back to the level of control “healthy” mice (Figure 4A). The changes in mRNA expression, however, were not reflected in the level of enzyme activity of CYP1A1/2 (Figure 5A). The same discrepancies between mRNA expression and enzyme activity were observed in the cases of Cyp2b10 and Cyp2c38. While mRNA expression of these genes was downregulated in all the groups treated with DSS, the enzyme activity of CYP2B and CYP2C were increased in all experimental groups compared to the control group (Figures 5B,C). Discrepancies between mRNA and the activity of CYP enzymes are common and have also been reported in previous reports using conventional and germ-free (GF) mice (Jourová et al., 2020b; Han et al., 2020; Togao et al., 2021). This phenomenon can be ascribed to different kinetics of mRNA and protein expression and post-transcriptional regulation. Moreover, CYP2B, CYP2C, and CYP3A have been reported to undergo regulation also by microRNA (Nakajima and Yokoi, 2011; Rieger et al., 2013).

In our study, DSS-induced colitis led to a significant increase of Cyp3a11 and Cyp3a13 mRNA expression as well as CYP3A enzyme activity in female murine liver (Figures 4E,F, Figure 5D). These results were contradictory to two previous studies where hepatic mRNA and protein expression and enzyme activity of CYP3A were decreased (Kawauchi et al., 2014; Kusunoki et al., 2014). This discrepancy may be caused by sex differences, as in these studies male mice were used, while female mice had been used in our study. Female mice have shown higher expression of Cyp3a11 and Cyp3a13 mRNA than males (Hernandez et al., 2009; Jourová et al., 2020b), and regulation of Cyp3a11 mRNA expression may be different during inflammation in female mice due to differences in growth hormone patterns (Holloway et al., 2006; Waxman and O'Connor, 2006).

Interestingly, the activity of CYP3A varied significantly between the groups where butyrate was administered in the two different time settings before colitis induction by the DSS (groups 3 and 4) (Figure 5D). On the basis of data known from the literature, it is possible to suggest a few possible explanations. The differences in time settings of butyrate administration in groups 3 and 4 may be crucial due to various levels of butyrate, as the ability of gut microbiota to synthesize butyrate as well, as butyrate uptake and utilization by colonocytes is impaired in IBD patients depending on the severity of inflammation (Laserna-Mendieta et al., 2018). Moreover, supplementation with butyrate has been shown to affect significantly the composition of gut microbiota (Lee et al., 2022), and previous studies on the gnotobiotic mice have revealed that the regulation of CYP3A is very sensitive to the interventions in the gut microbial community (Selwyn et al., 2016; Jourová et al., 2017). In another study, a strong correlation of some genus from the bacterial family *Clostridiaceae,* butyrate-producing bacterial family*,* and Cyp3a activity in mice was observed. In other words, a higher abundance of bacteria such as *Clostridium butyricum* producing high levels of butyrate may enhance CYP3A activity (Togao et al., 2021).

Altogether, these results indicate that the enzymes of this most abundant hepatic biotransformation enzyme family (CYP3A), metabolizing more than 50% of marketed drugs, may respond sensitively to gut microbiome-targeted interventions.

The obtained data indicate that not only inflammation but also butyrate administration may affect the enzyme activity of some CYPs and thus, modulate the response to many drugs metabolized by these important biotransformation enzymes. With regard to medications used in inflammatory diseases such as IBD and new microbiota-targeted therapeutic approaches, it is very important to deepen our knowledge of the effect of gut inflammation and followed therapeutic interventions on the ability of the organism to metabolize drugs.

In summary, these data clearly highlight that gut microbiome metabolite butyrate affects the inflammatory process in both the intestine and liver and hepatic drug metabolism by modulation of expression and enzyme activity of CYP enzymes. This gut–liver axis mediated through inflammation and microbiome-derived metabolites may have implications for health and diseases, and, most importantly, may affect the response to IBD therapy. Due to the increasing popularity of therapeutic interventions targeting the gut microbiome, further research in this field is needed.

## Data Availability

The original contributions presented in the study are included in the article/Supplementary Material, and further inquiries can be directed to the corresponding author.
